# Supplementary material for: Analysis of PPARγ Signaling Activity in Psoriasis
Source: Int J Mol Sci. 2021 Aug 10;22(16):8603. doi: 10.3390/ijms22168603 (PMC8395241; doi:10.3390/ijms22168603)
Supplement: Supplementary file 1 [file ijms-22-08603-s001.zip › Supplemental materials_Analysis of PPARg signaling activity in psoriasis/Pathway models/Models images and html files/Interleukin-17 and interleukin-22 signaling in psoriasis/9335.html]

MAP2K1


# Protein MAP2K1

|  |  |
| --- | --- |
| URN | urn:agi-llid:5604 |
| Total Entities | 0 |
| Connectivity | 3619 |
| Name | MAP2K1 |
| Description | mitogen-activated protein kinase kinase 1 |
| Notes | The protein encoded by this gene is a member of the dual specificity protein kinase family, which acts as a mitogen-activated protein (MAP) kinase kinase. MAP kinases, also known as extracellular signal-regulated kinases (ERKs), act as an integration point for multiple biochemical signals. This protein kinase lies upstream of MAP kinases and stimulates the enzymatic activity of MAP kinases upon wide variety of extra- and intracellular signals. As an essential component of MAP kinase signal transduction pathway, this kinase is involved in many cellular processes such as proliferation, differentiation, transcription regulation and development. [provided by RefSeq, Jul 2008] |
| Primary Cell Localization | Cytoplasm |
| Class | Protein kinase |

---

|  |  |
| --- | --- |
| Pathway | Alzheimer's Disease |
|  | Osteoarthritis |
|  | Skin Aging |
|  | Oncogene Induced Cellular Senescence |
|  | Melatonin Effects on Circadian Cycle |
|  | mTOR Signaling |
|  | mTOR Signaling Hyperfunction |
|  | High Fat Diet Activates cAMP Related Exocytosis in Synapse and Endocrine Cell |
|  | mTOR Signaling Activation by Taste Sensor Receptors (Rodent Model) |
|  | Leptin and CCK8 Activate Nodose Ganglia Neuron |
|  | Insulin Influence on Protein Synthesis |
|  | Insulin Influence on Glycogenesis |
|  | Insulin Synthesis in beta-Cell |
|  | Dopamine Receptors Signaling |
|  | Serotonin Receptors Signaling |
|  | Thyroid Hormones Common non-Genomic Effects in Hyperthyroidism |
|  | Norepinephrine Release Regulation |
|  | CRH Secretion Regulation |
|  | Insulin Signaling |
|  | Prolactin Production |
|  | POMC Secretion in adenohypophysis |
|  | Thrombopoietin Receptors Signaling in Platelet Maturation |
|  | Mineralcorticoids Secretion Regulation |
|  | T-Cell Dependent B-Cell Activation |
|  | T-Cell Independent B-Cell Activation |
|  | Natural Killer Cell Activation through C-Type Lectin-like Receptors |
|  | Natural Killer Cell Activation through ITAM-Containing Receptors |
|  | Natural Killer Cell Inhibitory Receptor Signaling |
|  | Natural Killer Cell Activation through ITSM-Containing Receptors |
|  | Natural Killer Cell Activation through Integrins and non-ITAM-Containing Receptors |
|  | T-Cell Maturation (Hypothesis) |
|  | Natural Killer Cell Activation |
|  | CD8+ T-Cell Activation |
|  | Th1-Cell Differentiation |
|  | Treg-Cell Differentiation |
|  | Th2-Cell Differentiation |
|  | T-Cell Receptor Signaling |
|  | CXC Chemokine Receptor Signaling |
|  | CC Chemokine Receptor Signaling |
|  | Plasmin Effects in Inflammation |
|  | Platelet Activation via Adhesion Molecules |
|  | Proplatelet Maturation |
|  | Platelet Activation via GPCR Signaling |
|  | Toll-like Receptor Independent Sterile Inflammation |
|  | Lipoxin A4/FPR2-Related Neutrophil Depression |
|  | MC1R in anti-Inflammatory Signaling |
|  | Eicosanoids in Myeloid Cell Activation |
|  | Leukotriene Effect on Airway Smooth Muscle Response |
|  | Neutrophil Activation via LTB4/Polyisoprenyl Phosphate Signaling |
|  | Basophil Activation |
|  | Eosinophil Survival by Cytokine Signaling |
|  | Eosinophil Activation |
|  | Mast-Cell Activation via IgE Signaling |
|  | Mast-Cell Activation without Degranulation through CYSLTR1/CYSLTR2 Signaling |
|  | Mast-Cells De Novo Synthesized Mediators via IgE Independent Signaling |
|  | Macrophage M1 Lineage |
|  | CD16/CD14 Proinflammatory Monocyte Activation |
|  | Macrophage M2-Related Phagocytosis |
|  | anti-Inflammatory Function of Macrophage M2 Lineage |
|  | Neutrophil Recruitment and Priming |
|  | Neutrophil Activation via FCGR3B |
|  | Neutrophil Chemotaxis |
|  | Neutrophil Activation via Adherence on Endothelial Cells |
|  | Positive Acute Phase Proteins Synthesis |
|  | Vascular Smooth Muscle Cell/Pericyte Migration and Proliferation |
|  | Vascular Endothelial Cell Activation by Blood Coagulation Factors |
|  | Vascular Endothelial Cell Activation by Growth Factors |
|  | Nociception Expression Targets Signaling |
|  | Vascular Motility |
|  | NPY1R -> CRH/POMC Production |
|  | NTSR1 Expression Targets |
|  | EDNRA/B -> Vascular Motility |
|  | ADRA1 -> Vasoconstriction |
|  | ADRA1A -> IL6 Production |
|  | ADRA2C/ADRB2 -> Vasoconstriction |
|  | ADRA2C/ADRB2 -> Synaptic Endocytosis |
|  | ADRB1 -> Prostaglandin Generation |
|  | ADRA1 -> Prostaglandin Generation |
|  | BDKRB1/2 -> Prostaglandin Generation |
|  | CNR1/2 -> IL1B/2/4/6/10 Production |
|  | DRD2 Expression Targets |
|  | DRD1/5 Expression Targets |
|  | GALR1/2/3 -> POMC/NPY Production |
|  | HRH2/4 -> IL6/10 Production |
|  | CHRM1 -> IL2 Production |
|  | CHRM1/2/3 -> Vascular Motility |
|  | OPRK -> Pain Perception |
|  | PTGIR -> IL6 Production |
|  | PTGER1/4 -> Vascular Motility |
|  | PTGER2/3 -> Inflammation-Related Expression Targets |
|  | HTR1 -> IL6 Production |
|  | HTR7 -> IL6 Production |
|  | HTR5 -> TNF Production |
|  | IL6 Expression Targets -> Nociception |
|  | IL1B Expression Targets -> Nociception |
|  | CHRNA7 -> NOS1 Production |
|  | GABA(B)R -> Postsynaptic Inhibition |
|  | NMDA Receptors -> Ca2+/CREB Activation/PGE2 Synthesis |
|  | P2RY11/13/14 -> IL8/10 Production |
|  | Luteinizing Hormone in Oocyte Meiotic Resumption |
|  | Melatonin in Cell Survival and Antioxidant Response |
|  | Circadian Clock Genes in Suprachiasmatic Nuclei Neurons |
|  | Vitamine D Deficite and Dentin Formation |
|  | non-Genomic Rapid Actions of Vitamin D in Vitamin D Biology |
|  | G0/G1 Phase Transition |
|  | Scavenger Receptor OLR1 in Inflammation Related Endothelial Dysfunction |
|  | RAGE/AGER and S100 Proteins in Cardiovascular Injury |
|  | Th1-Cells Activation and Proliferation in Atherosclerosis |
|  | Cardiomyocyte Hypertrophy |
|  | Hypertrophic Cardiomyopathy |
|  | Endothelin Signaling in Arterial Hypertension |
|  | Arterial Hypertension |
|  | Aldosterone Synthesis Acute Regulation |
|  | Endothelial Cell Dysfunction in Arterial Hypertension |
|  | Aldosterone Synthesis Chronic Regulation |
|  | Smooth Muscle Cell Dysfunction in Arterial Hypertension |
|  | TRPM4/6/7/8 Signaling in Arterial Hypertension (Hypothesis) |
|  | Smooth Muscle Cell Dysfunction in Pulmonary Hypertension |
|  | Myocardial Remodeling |
|  | Contraction Due Vasospasm |
|  | Sebocyte Proliferation in Acne Vulgaris |
|  | Inflammatory Reaction in Acne Vulgaris |
|  | Keratinocyte Proliferation in Acne Vulgaris |
|  | Hair Follicle Keratinocyte Apoptosis |
|  | Atopic Dermatitis Onset |
|  | Mast-Cells Activation in Atopic Dermatitis |
|  | Cell Cycle Dysregulation in Melanoma |
|  | Melanoma |
|  | Apoptosis Block in Melanoma Cell |
|  | MITF as a Regulator of Melanoma Cell Development |
|  | IL17 Signaling in Psoriasis |
|  | IL22 Induces Keratinocyte Proliferation in Psoriasis |
|  | Th0-Cell Aberrant Activation in Systemic Lupus Erythematosus |
|  | Th1-Cell Function in Systemic Lupus Erythematosus |
|  | B-Cell Activation in Systemic Lupus Erythematosus |
|  | Th2-Cell Function in Systemic Lupus Erythematosus |
|  | Acute Cytotoxic CD8+T-Cell Response against Melanocytes |
|  | Vitiligo |
|  | T-Cell Cytotoxic Response against Melanocytes in Vitiligo |
|  | Apoptosis of Melanocyte in Vitiligo |
|  | Humoral Immunity in Vitiligo |
|  | CD8+ T-Cell Response to Self-Determinants in Diabetes |
|  | Treg-Cell Function in Diabetes Mellitus Type 1 |
|  | Natural Killer T-Cell Roles in Diabetes Mellitus Type 1 |
|  | non-Suppressive Treg-Cell in Diabetes Mellitus Type 1 |
|  | TLR2 Signaling in Treg-Cell in Type 1 Diabetes (Animal Model) |
|  | Natural Killer Cell in Diabetes Mellitus Type 1 |
|  | Toll-like Receptors in beta-Cell |
|  | Peripheral Tolerance to Autoantigens Recession in Diabetes Mellitus Type 1 |
|  | Treg-Cell Activation in Diabetes Mellitus |
|  | Donohue Syndrome Progression (Hypothesis) |
|  | Advanced Glycation End Products Pathway in Diabetic Microangiopathy |
|  | Protein Kinase C in Diabetic Microangiopathy |
|  | Peripheral Tissue Microangiopathy |
|  | Tubular Cell Dysfunction in Progressive Diabetic Nephropathy |
|  | Endothelial Cell Dysfunction in Progressive Diabetic Nephropathy |
|  | Endothelial Cell Dysfunction in Early Diabetic Nephropathy |
|  | Mesangial Cell Dysfunction in Diabetic Nephropathy |
|  | Microangiopathy in Diabetic Neuropathy |
|  | Proliferative Diabetic Retinopathy |
|  | Adiponectin Synthesis Declines in Insulin Resistance |
|  | Hepatic Stellate Cells in non-Alcoholic Fatty Liver Disease |
|  | beta-Cell Destruction through Cytokines in Diabetes Mellitus |
|  | FOXO1 and SREBP-1C Role in beta-Cell Suppression (Rodent Model) |
|  | Hyperparathyroidism, Neonatal Severe |
|  | RET1 Mutation in Hyperparathyroidism |
|  | Hypothyrodism Associated Hyperprolactinemia |
|  | DRD2 Pharmacological Inhibition in Hyperprolactinemia |
|  | Ethanol Induced Efects in Hyperprolactinemia |
|  | Cardiovascular Effects in Hyperthyroidism |
|  | Thyroid Dysfunction |
|  | Graves Ophthalmopathy |
|  | GNRH Signaling Impairment in Hypogonadotrophic Hypogonadism |
|  | Myocyte Dysfunction in Hypoparathyroidism |
|  | Hypoparathyroidism, Primary |
|  | Thyroid Stimulating Hormone (TSH) Resistance in Congenital Hypothyroidism |
|  | Overt Hypothyroidism, Primary |
|  | Thyroid Stimulating Hormone (TSH) Secretion in Overt Hypothyroidism |
|  | Prolactinoma Overiew |
|  | CD4+T-Cell Response in Celiac Disease |
|  | CD8+ T-Cell Response in Celiac Disease |
|  | Celiac Disease |
|  | OipA Signaling in Helicobacter Pylori Infection |
|  | CagA Phosphorylation Dependent Signaling |
|  | EDN, NRG, NRTN, and GDNF/RET Signaling in Hirschsprung Disease |
|  | Th2-Cell Function in Ulcerative Colitis |
|  | T-Cell Cytotoxic Response in Ulcerative Colitis |
|  | Osteoclast Activation in Multiple Myeloma |
|  | IL6/IGF1/VEGFA Signaling in Multiple Myeloma |
|  | Hepatocellular Carcinoma |
|  | Growth Factor Signaling in Hepatocellular Carcinoma |
|  | Pancreatic Ductal Carcinoma |
|  | Growth Factor Signaling in Pancreatic Neoplasms |
|  | Pancreatic Neuroendocrine Tumors |
|  | TRPM7/8 in Pancreatic Adenocarcinoma |
|  | TGFBR Signaling in Pancreatic Neoplasms |
|  | Eosinophil Survival in Asthma |
|  | Il17 Signaling Related Neutrophilia in Asthma |
|  | Eosinophil Activation and Degranulation in Asthma |
|  | IL17F Signaling in Bronchial Epithelial Cell in Asthma |
|  | Airway Smooth Muscle Cell High Rate Proliferation |
|  | IgE Induces Airway Smooth Muscle Cell Proliferation |
|  | Goblet-Cell Related Mucus Secretion in Asthma |
|  | Neutrophil Degranulation via FPR1/IL8 |
|  | Basophil Activation in Asthma |
|  | Mast-Cell Activation in Asthma |
|  | Antigen-Presenting Cell Role in Asthma |
|  | Th2-Cell Response in Asthma |
|  | Mucin Production in Goblet Airway Epithelial Cells |
|  | Osteoclast Activation in Gout |
|  | TNF and IL1B Induce Metalloproteinase Synthesis in Osteoarthritis |
|  | IL1B Induced Arthralgia |
|  | Steroidogenesis Impairement in Polycystic Ovary Syndrome |
|  | Ovulation Block |
|  | Polycystic Ovary Syndrome |
|  | FSH Action in Polycystic Ovary Syndrome |
|  | Keratinocyte Activation in Psoriatic Arthritis |
|  | PTPN22 Role in Rheumatoid Arthritis |
|  | Synovial Fibroblast Proliferation in Rheumatoid Arthritis |
|  | Osteoclast Activation in Rheumatoid Arthritis |
|  | B-Cells Function in Systemic Scleroderma |
|  | Th2-Cells Function in Systemic Scleroderma |
|  | Skin Fibrosis |
|  | WHIM Syndrome |
|  | Dentin Formation Disruption |
|  | CCR5 Signaling in Macrophages |
|  | CD8+ T-Cell Action Impairment in HIV Type 1 Infection |
|  | CD4+ T-Cell Function Decline in HIV |
|  | CD4+ T-Cell Death in HIV |
|  | Estrogen Effects on Pregnancy Gingivitis |
|  | IL1 Receptor Antagonist (IL1RN) Deficiency (DIRA) |
|  | Cowden Syndrome |
|  | Gluten Impact on Neuronal System (Hypothesis) |
|  | Osteoblast Function Decline in Osteoporosis |
|  | Osteoclast Activation in Postmenopause |
|  | BDNF Upregulation Triggered by Seizures |
|  | Apoptosis and Immediate Early Gene Activation in Epileptiform Disorders |
|  | Synaptic Exocytosis in Dementia |
|  | Glioblastoma, Primary |
|  | Glioblastoma, Secondary |
|  | Astrocytoma |
|  | Growth Factor Signaling in Neuroblastoma |
|  | Neuroblastoma |
|  | ALK Associated Neuroblastoma |
|  | Proteins Involved in Breast Cancer Related to ESR1 Signaling Pathway |
|  | Proteins Involved in Breast Cancer Related to ERBB2/VEGFR/Akt Signaling Pathway |
|  | ERBB/VEGFR/Akt Signaling in Breast Cancer |
|  | IGF1R/AKT Signaling in Breast Cancer |
|  | ESR1/ERBB Positive Luminal Breast Cancer |
|  | Proteins Involved in Breast Cancer Related to IGF1R/Akt Signaling Pathway |
|  | Breast Cancer |
|  | ESR1 Signaling in Breast Cancer |
|  | Endometrial Cancer |
|  | Endometrioid Endometrial Cancer |
|  | Clear Cell Endometrial Cancer and Papillary Serous Endometrial Cancer |
|  | Angiogenesis in Endometriosis |
|  | Coagulation Factors and PAR1/2 Receptors (F2R/F2RL1) in Endometriosis |
|  | Leptin Role in Endometriosis |
|  | Endometrioid Ovarian Carcinoma |
|  | Clear Cell Ovarian Carcinoma |
|  | EGFR and PTAFR Signaling in Ovarian Cancer |
|  | High-Grade Serous Ovarian Carcinoma |
|  | Low-Grade Serous Ovarian Carcinoma |
|  | Ovarian Cancer |
|  | Mucinous Ovarian Carcinoma |
|  | MIF Role in Cholesteatoma |
|  | HMGB1/RAGE Signaling in Middle Ear Cholesteatoma |
|  | Subepithelial Fibroblasts in Middle Ear Cholesteatoma |
|  | Cholesteatoma |
|  | HPV E5 Regulates EDNRA and EGFR Signaling Pathway in Keratinocytes |
|  | TRPM8 Effects in Prostate Cancer (Hypothesis) |
|  | Proteins Involved in Cataract |
|  | Steroids Induced Cataract |
|  | VEGFA in Neovascular Glaucoma |
|  | PGF2-alpha Effect on Treatment |
|  | Cytokines Trigger Otitis Media |
|  | MUC5AC Expression by NTHi and S. Pneumoniae |
|  | Alveolar Macrophage Negative Regulation Declined in COPD |
|  | VEGFA Low Expression Level in Chronic Bronchitis |
|  | VEGFA High Expression Level in Pulmonary Emphysema |
|  | Alveolar Epithelial Cell Dysfunction in COPD |
|  | Mucin Hyperproduction in Goblet and Mucous Cells |
|  | Polycystins Mutation Effects in Cystic Kidney Disease |
|  | Plasmin Role in Glomerulonephritis |
|  | Endothelial Cell Dysfunction in Glomerulonephritis |
|  | Mesangial Cell Dysfunction in Glomerulonephritis |
|  | Interstitial Fibroblasts in Pyelonephritis |
|  | Sertoli Cells Dysfunction Causes Male Infertility |
|  | CD38/CD3 -> JUN/FOS/NF-kB Signaling in T-cell Proliferation |
|  | CD38/CD19 -> JUN/FOS/NF-kB Signaling in B-cell Proliferation |
|  | CD38 -> cADPR/Calcium Signaling |
|  | CD157 -> cADPR/Calcium Signaling |
|  | S100P Expression Target |
|  | HMGB1 Expression Targets |
|  | TTR Expression Targets |
|  | S100A Expression Targets |
|  | S100B Expression Targets |
|  | ANGPT1 -> CREB/CREBBP Expression Targets |
|  | ANGPT2 -> AP-1/CREBBP/MYC Expression Targets |
|  | AREG -> CREB/CREBBP Expression Targets |
|  | AREG -> NCOR2 Expression Targets |
|  | AREG -> SMAD1 Expression Target |
|  | AREG -> HIF1A Expression Targets |
|  | AREG -> AP-1 Expression Targets |
|  | Noradrenaline/Gs Expression Targets |
|  | Dopamine/Gs Expression Targets |
|  | Serotonin/Gs Expression Targets |
|  | Noreadrenaline/Gq Expression Targets |
|  | Epinephrine/Gs Expression Targets |
|  | Acetylcholine Expression Targets |
|  | Serotonin/Gq Expression Targets |
|  | Epinephrine/Gq Expression Targets |
|  | Dopamine/Gi Expression Targets |
|  | Aryl Hydrocarbon Receptor Genomic and non-Genomic Signaling |
|  | B-Cell Receptor -> AP-1 Signaling |
|  | BTC -> AP-1/ATF/CREB Expression Targets |
|  | BTC -> EP300/ETS/ETV/SP1 Expression Targets |
|  | GLP1R -> ARRB1 Signaling |
|  | AGTR1 -> ARRB1/ARRB2 Signaling |
|  | SELE -> ELK-SRF Signaling |
|  | CD19 Expression Targets |
|  | CD81 -> AP-1 Expression Targets |
|  | CD247 Expression Targets |
|  | gamma Globulins Expression Targets |
|  | FCGR3A Expression Targets |
|  | CD19/CR2 Expression Targets |
|  | CD72 -> CREB/CREBBP Expression Targets |
|  | CD8 -> ATF/CREB/CREBBP Expression Targets |
|  | CD80 -> NF-kB Expression Targets |
|  | CD86 -> NF-kB Expression Targets |
|  | CXCL6 Expression Targets |
|  | CXCL1 Expression Targets |
|  | CXCL2 Expression Targets |
|  | CXCL3 Expression Targets |
|  | CXCL5 Expression Targets |
|  | CHRNA7 -> CREB Signaling |
|  | Clozapine Induced Granulocytopenia |
|  | EGF -> HIF1A Expression Targets |
|  | EGF -> AP-1/ATF Expression Targets |
|  | EGF -> CREB/CREBBP/ELK/SRF/MYC Expression Targets |
|  | EGF -> TP53 Expression Targets |
|  | EGF -> NCOR2 Expression Target |
|  | EGFR -> NCOR2 Signaling |
|  | EGFR/ERBB2 -> TP53 Signaling |
|  | ERBB2/3 -> EP300/ETS/ETV/SP1 Signaling |
|  | EGFR -> SMAD1 Signaling |
|  | EGFR/ERBB2 -> HIF1A Signaling |
|  | EGFR -> AP-1/ATF2 Signaling |
|  | EGFR -> AP-1/CREB/ELK/SRF/MYC Signaling |
|  | EREG -> CREB Expression Target |
|  | EREG -> EP300/SP1 Expression Targets |
|  | EREG -> HIF1A Expression Target |
|  | EREG -> AP-1/ATF Expression Targets |
|  | Erythropoietin -> AP-1/MYC/CREB Expression Targets |
|  | Erythropoietin -> ELK/SRF Expression Targets |
|  | PECAM1 Expression Targets |
|  | NCAM1 Expression Targets |
|  | F2 -> AP-1/CREB/ELK/SRF/SP1 Expression Targets |
|  | PLG -> AP-1/CREB/ELK/SRF/SP1 Expression Targets |
|  | FGF9 -> AP-1/CREB/MYC Expression Targets |
|  | FGF10 -> AP-1/CREB/CREBBP/MYC Expression Targets |
|  | FGF1 -> AP-1/CREB/ELK/SRF/MYC Expression Targets |
|  | FGF1 -> NCOR2 Expression Target |
|  | FGF7 -> RUNX Expression Targets |
|  | FGF8 -> AP-1/CREB/MYC Expression Targets |
|  | FGF1 -> RUNX Expression Targets |
|  | FGF18 -> AP-1/CREB Expression Targets |
|  | FGF23 -> NCOR2 Expression Targets |
|  | FGF8 -> RUNX Expression Targets |
|  | FGF9 -> RUNX Expression Targets |
|  | FGF2 -> NCOR2 Expression Targets |
|  | FGF4 -> AP-1/MYC Expression Targets |
|  | FGF7 -> AP-1/CREB/CREBBP/MYC Expression Targets |
|  | FGF2 -> RUNX Expression Targets |
|  | FGF2 -> AP-1/CREB/CREBBP/ELK/SRF/MYC Expression Targets |
|  | FGFR -> RUNX2 Signaling |
|  | FGFR -> AP-1/CREB/CREBBP/ELK/SRF/MYC Signaling |
|  | GCG Expression Targets |
|  | ADCYAP1 Expression Targets |
|  | FSHR Expression Targets |
|  | Glutamate/Gq Expression Targets |
|  | VIP Expression Targets |
|  | GH1/GHR -> NF-kB/ELK/SRF/MYC Expression Targets |
|  | CSH1/GHR Expression Targets |
|  | HBEGF -> AP-1/ATF Expression Targets |
|  | HBEGF -> TP53 Expression Targets |
|  | HBEGF -> EP300/ETS/ETV/SP1 Expression Targets |
|  | HBEGF -> HIF1A Expression Targets |
|  | HBEGF -> CREB/MYC Expression Targets |
|  | HGF -> AP-1/CREB/ELK/SRF/MYC Expression Targets |
|  | HGFR -> AP-1/CREB/MYC Signaling |
|  | TAM Receptor Signaling Activation in Cancer |
|  | Proteins with Altered Expression in Cancer-Associated Sustaining of Proliferative Signaling |
|  | Genes with Mutations in Cancer-Associated Sustaining of Proliferative Signaling |
|  | G0/G1 Cell Cycle Phase Transition Activation in Cancer |
|  | RAS/RAF/MAPK Signaling Activation in Cancer |
|  | RAS/RAF/MAPK Signaling Activation by Blocking of Tumor Suppressors |
|  | TGFB Signaling activation by Blocking of Tumor Suppressors |
|  | HIF1A in Vasculogenic Mimicry of Cancer |
|  | VEGFA Dependent Angiogenesis in Cancer |
|  | VEGF Independent Angiogenesis in Cancer |
|  | Integrins in Cancer Cell Motility, Invasion and Survival |
|  | CDH2 Activation Promotes Cancer Cell Migration and Survival |
|  | TGFB Family in Epithelial to Mesenchymal Transition in Cancer |
|  | Treg Cells Promote Immunosuppression in Cancer Immune Escape |
|  | EZH2 Methyltransferase Role in Cancer |
|  | HCAR1/HCAR2 -> MAPK Signaling |
|  | IGF2 -> HIF1A/MYC Expression Targets |
|  | IGF1 -> ELK/SRF/HIF1A/MYC/SREBF Expression Targets |
|  | Insulin -> ELK/SRF/HIF1A/MYC/SREBF Expression Targets |
|  | AGER -> CREB/SP1 Signaling |
|  | NCAM1 -> CREB/ELK/SRF/MYC Signaling |
|  | CD19 -> JUN/ELK Signaling |
|  | PECAM -> SP1 Signaling |
|  | FcIgER -> ELK-SRF Signaling |
|  | ICAM1 -> AP-1/CREB/ELK/SRF Signaling |
|  | IGF1R -> ELK/SRF/HIF1A/MYC/SREBF Signaling |
|  | InsulinR -> ELK/SRF/SREBF Signaling |
|  | FibronectinR -> AP-1/ELK/SRF/SREBF Signaling |
|  | Fibronectin Expression Targets |
|  | CTGF -> AP-1/CREB/MYC Expression Targets |
|  | CTGF -> NCOR2 Expression Target |
|  | ICAM1 Expression Targets |
|  | IL6R -> CEBP/ELK/SRF Signaling |
|  | IL2R -> ELK/SRF/MYC Signaling |
|  | IL1R -> NF-kB Signaling |
|  | IL4R -> ELK/SRF/HMGY Signaling |
|  | IL16 -> AP-1 Expression Targets |
|  | IL6 Expression Targets |
|  | IL4 Expression Targets |
|  | IL2 Expression Targets |
|  | UrokinaseR -> ELK/SRF Signaling |
|  | PTPRC -> BCL6 Expression Targets |
|  | Leptin -> ELK/SRF Expression Targets |
|  | Prostaglandin F Expression Targets |
|  | Thromboxane A2 Expression Targets |
|  | LPA Expression Targets |
|  | PAF/Gq -> AP-1/ATF1/CREB/ERK/SRF Expression Targets |
|  | Dronabinol/Anandamide Expression Targets |
|  | Morphine Expression Targets |
|  | S1P Expression Targets |
|  | PGE1 Expression Targets |
|  | MTNR1 Signaling |
|  | NRG1 -> EP300/ETS/ETV/SP1 Expression Targets |
|  | NRG1 -> CREB/CREBBP/ELK/SRF/MYC Expression Targets |
|  | NRG1 -> TP53 Expression Target |
|  | NRG1 -> AP-1/ATF Expression Targets |
|  | NRG1 -> HIF1A Expression Target |
|  | NGF -> AP-1/TP53/MYC Expression Targets |
|  | NGF -> FOXO/MYCN/ELK-SRF Expression Targets |
|  | BDNF Expression Targets |
|  | NTF3 Expression Targets |
|  | NTF4 Expression Targets |
|  | NGF -> CREB/CEBPB/MEF2A Expression Targets |
|  | NOTCH -> TCF3 Signaling |
|  | NOTCH Expression Targets |
|  | Adenosine Expression Targets |
|  | OR1A1 -> GNAS Signaling |
|  | PDGFB -> AP-1/CREB/MYC Expression Target |
|  | PDGFC -> CREB Expression Target |
|  | PDGFD -> AP-1 Expression Targets |
|  | PDGF -> AP-1/CREB/CREBBP/MYC Expression Targets |
|  | KIT -> MITF Signaling |
|  | PDGFR -> AP-1/MYC Signaling |
|  | AVP/Gs -> CREB/ELK/SRF/AP-1/EGR Expression Targets |
|  | GNRH1 Expression Targets |
|  | NTS Expression Targets |
|  | GAST Expression Targets in GAST Expression Targets |
|  | AGT -> TP53 Expression Targets |
|  | GNRH2 Expression Targets |
|  | OXT Expression Targets |
|  | POMC Expression Targets |
|  | CCL11 Expression Targets |
|  | EDN1 Expression Targets |
|  | AGT -> CREB Expression Targets |
|  | NPY Expression Targets |
|  | IFNA1/Gq Expression Targets |
|  | AGT -> ELK/SRF Expression Targets |
|  | TAC1 Expression Targets |
|  | EDN3 Expression Targets |
|  | AVP/Gq -> CREB/ELK/SRF/AP-1/EGR Expression Targets |
|  | PRL/GHR -> NF/kB/ELK/SRF/MYC Expression Targets |
|  | PDCD1 -> AP-1 Expression Targets |
|  | PTPRC -> BCL6 Signaling |
|  | AngiopoietinR -> AP-1 Signaling |
|  | GDNF -> HSF1 Signaling |
|  | EphrinB -> MAPK/JUN/FOS Signaling |
|  | NTRK -> AP-1/CREB/ELK/SRF/MYC/SMAD3/TP53 Signaling |
|  | GAS6 -> AP-1/CREB Expression Targets |
|  | FIGF -> AP-1 Expression Target |
|  | FIGF -> NCOR2 Expression Target |
|  | KITLG -> AP-1/CREB/CREBBP/MYC Expression Targets |
|  | GDNF -> HSF1 Expression Targets |
|  | CSF1 -> AP-1/CREB/CREBBP/MYC Expression Targets |
|  | PGF -> AP-1/CREB/CREBBP/MYC Expression Targets |
|  | FLT3LG -> AP-1/CREB/CREBBP Expression Targets |
|  | Lysophosphatidic Acid/LPARs Signaling |
|  | Androgen Receptor non-Genomic Signaling |
|  | FOXO1 Signaling |
|  | MERTK Signaling |
|  | Ras Signaling |
|  | TGF-beta Signaling |
|  | AMPK Signaling |
|  | AXL Receptor Tyrosine Kinase Signaling |
|  | KRAS Signaling |
|  | VEGF Signaling |
|  | Estrogens/ESR1 non-Genomic Signaling |
|  | EGFR Signaling |
|  | HRAS Signaling |
|  | ERK/MAPK Canonical Signaling |
|  | TCR -> AP-1 Expression Targets |
|  | T-Cell Receptor -> AP-1 Signaling |
|  | TGFA -> TP53 Expression Targets |
|  | TGFA -> HIF1A Expression Targets |
|  | TGFA/AP-1/ATF Expression Targets |
|  | TGFA -> CREB/CREBBP/ELK-SRF/MYC Expression Targets |
|  | FASLG Expression Targets |
|  | TNFSF10 Expression Targets |
|  | LTA Expression Targets |
|  | TNFSF14 Expression Targets |
|  | TNF -> AP-1 Expression Targets |
|  | CD72 -> AP-1 Expression Targets |
|  | CD40LG -> NF-kB/ELK/SRF -> CREB/NFATC Expression Targets |
|  | TNFSF13 Expression Targets |
|  | TNFSF13B Expression Targets |
|  | TNF -> ELK-SRF Expression Targets |
|  | TNF -> CREB Expression Targets |
|  | TNF -> TP53/ATF Expression Targets |
|  | EDA Expression Targets |
|  | EctodysplasinR -> AP-1 Signaling |
|  | TNFR -> CREB/ELK-SRF Signaling |
|  | TNFR -> AP-1/ATF/TP53 Signaling |
|  | NGFR -> AP-1/CEBPB/CREB/ELK/SRF/TP53 Signaling |
|  | TNFRSF6 -> HSF1 Signaling |
|  | TNFRSF1A -> AP-1/ATF/TP53 Signaling |
|  | TNFRSF1A -> CREB/ELK-SRF Signaling |
|  | Thrombopoietin -> AP-1/CREB/CREBBP/MYC Expression Targets |
|  | Thrombopoietin -> SP1 Expression Targets |
|  | IL1B -> NO Expression Targets |
|  | IL1B -> PGE2 Expression Targets |
|  | IL1B Expression Targets |
|  | IL1A Expression Targets |
|  | ROS in Angiotensin Mediated Cardiovascular Remodeling and Hypertrophy |
|  | PLAU -> ELK-SRF/AP-1 Expression Targets |
|  | VEGFA -> ATF/CREB/ELK-SRF Expression Targets |
|  | VEGFC -> ATF Expression Target |
|  | VEGFA -> AP-1/CREBBP/MYC Expression Targets |
|  | VEGFA -> NCOR2 Expression Target |
|  | VEGFR -> AP-1/CREB/MYC Signaling |
|  | VEGFR -> ATF/CREB/ELK-SRF Signaling |
|  | pathway 3 |
|  | ace2 |
|  | Model of PPARG signaling in psoriasis |
|  | Model of PPARG related pathways in psoriasis (short version) |
|  | 1\_Inflammation in keratinocytes in the pathology of acne |
|  | 2\_Hyperseborrhea in acne vulgaris |
|  | 2\_Acute phase initiation in atopic dermatitis |
|  | 2\_Interleukin-17 and interleukin-22 signaling in psoriasis |
|  | 1\_The onset of atopic dermatitis |
|  | 1\_TNF and IL1B provoke ECM degradation in osteoarthritis |
|  | 2\_Osteoblast-mediated bone demineralization |
|  | 1\_Formation of cysts in polycystic kidney disease |
|  | 2\_The role of endothelial cells in pyelonephritis |
|  | 3\_The role of interstitial fibroblasts in pyelonephritis |
|  | 1\_Local estrogen production stimulates ectopic endometrium proliferation |
|  | 3\_Enhanced angiogenesis in endometriosis |
|  | 2\_Impaired steroidogenesis in PCOS |
|  | 1\_Disruption of luteinizing hormone and follicle-stimulating hormone secretion in PCOS |
|  | 2\_Pathogens stimulate mucins expression in the middle ear |
|  | 2\_Oxidized LDL causes endothelial dysfunction in atherosclerosis |
|  | 2\_Cardiomyocyte hypertrophy |
|  | 3\_Dysfunction of smooth muscle cells in hypertension |
|  | 1\_Rise in aldosterone synthesis in adrenal zona glomerulosa cells |
|  | 2\_The intense proliferation and contractile activity of smooth muscle cells in pulmonary hypertension |
|  | 1\_Cigarette smoke causes dysfunction and production of inflammatory mediators in alveolar epithelial cells |
|  | 3\_Role of alveolar macrophages in pulmonary tissue destruction in COPD |
|  | 1\_Th2 cell response in asthma |
|  | 3\_1\_Mucus accumulation in asthma |
|  | 2\_1\_Eosinophilia and neutrophilia in asthma: eosinophil migration and activation |
|  | 2\_2\_Eosinophilia and neutrophilia in asthma: eosinophil apoptosis |
|  | 4\_2\_Airway smooth muscle cells proliferation and airway tissue remodeling |
|  | 4\_Mucin hyperproduction in goblet and mucous cells in COPD |
|  | 1\_CD4+ T cell and epithelial cell response in celiac disease |
|  | 2\_CD8+ T-cell response and mucosal damage in celiac disease |
|  | 1\_1\_Insulin synthesis and secretion: insulin processing and storage |
|  | 4\_1\_Insulin resistance: insulin signaling |
|  | 3\_Islet beta-cell destruction in T1D |
|  | 1\_Microangiopathy in diabetic neuropathy |
|  | 2\_1\_General pathways of thyroid hormone signaling |
|  | Model of PPARG signaling in psoriais (tested) |
|  | public list |
|  | New Pathway |
|  | HRAS->RAF->MAPK1/3 |
|  | KRAS->RAFs->MAPK1/3 |
|  | NRAS->RAFs->MAPK1/3 |
|  | mapk1 |
|  | New Pathway |
|  | mapk1 protein modificators |
|  | Interleukin-17 and interleukin-22 signaling in psoriasis |

---

|  |  |
| --- | --- |
| Group | Proteins Involved in Cowden Syndrome |
|  | Genes Associated with Syndromic Epilepsy |

---

|  |  |
| --- | --- |
| Source | GO component |
|  | curated |

---

|  |  |
| --- | --- |
| MedScan ID | 5604 |

---

|  |  |
| --- | --- |
| LocusLink ID | 5604 |
|  | 26395 |
|  | 170851 |
|  | 336276 |
|  | 19101 |
|  | 336209 |
|  | 533199 |
|  | 406728 |
|  | 326395 |

---

|  |  |
| --- | --- |
| Alias | MAPKK I |
|  | MPK I |
|  | MAPK kinase I |
|  | MAP kinase kinase 1 |
|  | MAP kinase kinase I |
|  | ERK kinase 1 |
|  | MAP kinase/Erk kinase I |
|  | MAP kinase kinase 1 , splice form A |
|  | MAP2K1 |
|  | MAPK kinase 1 |
|  | MAP kinase/Erk kinase 1 |
|  | PRKMK I |
|  | ERK activator kinase I |
|  | mitogen activated protein kinase kinase 1 |
|  | OTTMUSP00000018355 |
|  | MPK1 |
|  | MAPK/ERK kinase I |
|  | protein kinase, mitogen-activated, kinase 1 (MAP kinase kinase 1) |
|  | Map2k I |
|  | protein kinase, mitogen activated, kinase 1, p45 |
|  | MKK I |
|  | PRKMK1 |
|  | MPK1\_HUMAN |
|  | MKK1 |
|  | ERK activator kinase 1 |
|  | ERK kinase I |
|  | MAPK/ERK kinase 1 |
|  | Dual specificity mitogen-activated protein kinase kinase I |
|  | MAPKK1 |
|  | mitogen-activated protein kinase kinase I |
|  | Dual specificity mitogen-activated protein kinase kinase 1 |
|  | MEK1 |
|  | MEK I |
|  | CFC3 |
|  | MAPKK 1 |
|  | MEK 1 |
|  | MEKK1 |
|  | mitogen-activated protein kinase kinase 1 |
|  | PRKMK1s |
|  | MAPKK1s |
|  | Edpm9 |
|  | PRKMK Is |
|  | Map3k1 |
|  | mitogen-activated |
|  | Estrogen-dependent pituitary mass QTL 9 |
|  | MAPKKK1 |
|  | protein kinase, mitogen-activated, kinase I |
|  | oestrogen-dependent pituitary mass qtl, chromosome 9 |
|  | MAPKK Is |
|  | kinase 1 |
|  | Estrogen-dependent pituitary mass QTL, Chromosome 9 |
|  | kinase 1 (MAP kinase kinase 1) |
|  | Estrogen-dependent pituitary mass QTL IX |
|  | protein kinase, mitogen-activated, kinase 1 |
|  | protein kinase |
|  | kinase, mitogen activated, kinase 1, p |
|  | Mekk |
|  | oestrogen-dependent pituitary mass qtl, chromosome IX |
|  | MEK1(MAP2K1) |

---

|  |  |
| --- | --- |
| EC Number | 2.7.12.2 |
|  | 2.7.1.- |

---

|  |  |
| --- | --- |
| GO ID | 0005524 |
|  | 0004708 |
|  | 0008022 |
|  | 0047485 |
|  | 0004672 |
|  | 0043539 |
|  | 0004674 |
|  | 0004712 |
|  | 0004713 |
|  | 0097110 |
|  | 0060020 |
|  | 0070371 |
|  | 0000165 |
|  | 0000187 |
|  | 0032147 |
|  | 0007050 |
|  | 0048870 |
|  | 0090398 |
|  | 0021697 |
|  | 0006935 |
|  | 0060502 |
|  | 0060324 |
|  | 0007507 |
|  | 0030216 |
|  | 0060711 |
|  | 0008285 |
|  | 0010629 |
|  | 0030182 |
|  | 0018107 |
|  | 0018108 |
|  | 0060674 |
|  | 0070374 |
|  | 0050772 |
|  | 0010628 |
|  | 1903800 |
|  | 0071902 |
|  | 0045893 |
|  | 0006468 |
|  | 0090170 |
|  | 0048679 |
|  | 2000641 |
|  | 0032872 |
|  | 0007165 |
|  | 0023014 |
|  | 0031098 |
|  | 0048538 |
|  | 0030878 |
|  | 0060440 |
|  | 0005794 |
|  | 0005737 |
|  | 0005829 |
|  | 0005769 |
|  | 0005783 |
|  | 0005925 |
|  | 0005770 |
|  | 0005815 |
|  | 0005739 |
|  | 0005634 |
|  | 0005886 |
|  | 0017016 |
|  | 0016301 |
|  | 0031435 |
|  | 0000166 |
|  | 0019901 |
|  | 0016740 |
|  | 0048313 |
|  | 0008283 |
|  | 0042593 |
|  | 0060425 |
|  | 0032402 |
|  | 0000278 |
|  | 0034111 |
|  | 1903298 |
|  | 0048812 |
|  | 0016310 |
|  | 2001171 |
|  | 0043547 |
|  | 0046579 |
|  | 0010508 |
|  | 0045597 |
|  | 0030335 |
|  | 0045933 |
|  | 0032968 |
|  | 0051291 |
|  | 0070372 |
|  | 0098696 |
|  | 0003056 |
|  | 0048678 |
|  | 0051384 |
|  | 0006979 |
|  | 0070328 |
|  | 0047496 |
|  | 0030424 |
|  | 0005938 |
|  | 0005856 |
|  | 0030425 |
|  | 0032839 |
|  | 0098978 |
|  | 0016020 |
|  | 0005874 |
|  | 0043204 |
|  | 0048471 |
|  | 0014069 |
|  | 0035556 |
|  | 0004702 |
|  | 0004728 |
|  | 0098779 |
|  | 0006928 |
|  | 0035897 |
|  | 0070062 |
|  | 0007067 |
|  | 0001932 |
|  | 0042981 |
|  | 0007346 |
|  | 0038095 |
|  | 0002755 |
|  | 0002756 |
|  | 0007265 |
|  | 0035666 |
|  | 0000186 |
|  | 0007411 |
|  | 0007173 |
|  | 0008543 |
|  | 0045087 |
|  | 0008286 |
|  | 0048011 |
|  | 0009405 |
|  | 0032320 |
|  | 0007264 |
|  | 0051403 |
|  | 0034166 |
|  | 0034134 |
|  | 0034138 |
|  | 0034142 |
|  | 0034146 |
|  | 0034162 |
|  | 0038123 |
|  | 0038124 |
|  | 0002224 |
|  | 0048010 |
|  | 0016772 |
|  | 0035335 |
|  | 0005816 |
|  | 0008063 |
|  | 0034130 |
|  | 0033267 |
|  | 0007243 |
|  | 0005625 |
|  | 0008152 |
|  | 0016311 |
|  | 0010149 |
|  | 0005515 |
|  | 0043406 |
|  | 0005575 |

---

|  |  |
| --- | --- |
| KEGG ID | hsa:5604 |
|  | mmu:26395 |
|  | rno:170851 |

---

|  |  |
| --- | --- |
| MedLine Reference | 17461449 |
|  | 8462694 |
|  | 8131746 |
|  | 16481589 |
|  | 12270934 |
|  | 15557124 |
|  | 12594221 |
|  | 8297798 |
|  | 1381507 |
|  | 15890671 |
|  | 10489373 |
|  | 12832465 |
|  | 10780709 |
|  | 15302887 |
|  | 9779826 |
|  | 11823456 |
|  | 8168826 |
|  | 9325171 |
|  | 15069060 |
|  | 10395327 |
|  | 11579140 |
|  | 12792650 |
|  | 16424016 |
|  | 12466851 |
|  | 17498664 |
|  | 12697810 |
|  | 15538402 |
|  | 15572374 |
|  | 8626767 |
|  | 1411546 |
|  | 11217851 |
|  | 15021912 |
|  | 11076861 |
|  | 15304090 |
|  | 9149902 |
|  | 12663500 |
|  | 9558095 |
|  | 12807433 |
|  | 14672918 |
|  | 10570282 |
|  | 8388392 |
|  | 15757891 |
|  | 11134045 |
|  | 7914033 |
|  | 8529659 |
|  | 15657353 |
|  | 15917294 |
|  | 1281467 |
|  | 17182854 |
|  | 12370306 |
|  | 15240686 |
|  | 16861903 |
|  | 12689928 |
|  | 15020233 |
|  | 14656894 |
|  | 17366577 |
|  | 12609978 |
|  | 11604401 |
|  | 17192257 |
|  | 12244094 |
|  | 15701678 |
|  | 7969158 |
|  | 17197157 |
|  | 12063167 |
|  | 14627958 |
|  | 10725249 |
|  | 9440696 |
|  | 7957875 |
|  | 8602223 |
|  | 16737746 |
|  | 8602221 |
|  | 15353548 |
|  | 15292274 |
|  | 16086581 |
|  | 10982368 |
|  | 10209122 |
|  | 12695496 |
|  | 7601337 |
|  | 17038630 |
|  | 11841548 |
|  | 15657590 |
|  | 15615716 |
|  | 12167619 |
|  | 15665520 |
|  | 10523642 |
|  | 17054908 |
|  | 15979847 |
|  | 11042159 |
|  | 14594209 |
|  | 8157000 |
|  | 12531514 |
|  | 11409852 |
|  | 11044099 |
|  | 12917419 |
|  | 15469737 |
|  | 16199873 |
|  | 16463380 |
|  | 15118098 |
|  | 15284233 |
|  | 15782199 |
|  | 17490600 |
|  | 11352917 |
|  | 9841871 |
|  | 16439621 |
|  | 9621077 |
|  | 10828601 |
|  | 17603482 |
|  | 8406028 |
|  | 8226933 |
|  | 9223324 |
|  | 10997882 |
|  | 10806207 |
|  | 15159407 |
|  | 15145949 |
|  | 9852579 |
|  | 9733512 |
|  | 12875843 |
|  | 12876277 |
|  | 12788225 |
|  | 16686604 |
|  | 11266467 |
|  | 15004527 |
|  | 14563325 |
|  | 16913865 |
|  | 15753041 |
|  | 11226259 |
|  | 16820947 |
|  | 17183546 |
|  | 16931773 |
|  | 11104681 |
|  | 12663662 |
|  | 9553107 |
|  | 10536014 |
|  | 16761309 |
|  | 9465908 |
|  | 10757792 |
|  | 9208850 |
|  | 12839928 |
|  | 12482669 |
|  | 16887817 |
|  | 15489334 |
|  | 15509711 |
|  | 11222509 |
|  | 8385802 |
|  | 11260070 |
|  | 12975377 |
|  | 7624324 |
|  | 15737734 |
|  | 17419998 |
|  | 15297310 |
|  | 17260965 |
|  | 16239230 |
|  | 16141073 |
|  | 16141072 |
|  | 10912793 |
|  | 12456688 |
|  | 7731720 |
|  | 16728640 |
|  | 10748187 |
|  | 12532330 |
|  | 17396120 |
|  | 9806642 |
|  | 9155017 |
|  | 11535599 |
|  | 15647843 |
|  | 10917603 |
|  | 8394352 |
|  | 1379797 |
|  | 14963006 |
|  | 11278389 |
|  | 11891225 |
|  | 16313339 |
|  | 12401521 |
|  | 10799874 |
|  | 16148006 |
|  | 15543157 |
|  | 15305288 |
|  | 15782137 |
|  | 16957420 |
|  | 10716983 |
|  | 12032872 |
|  | 12612059 |
|  | 8380494 |
|  | 70317 |
|  | 12612072 |
|  | 17202850 |
|  | 9563949 |
|  | 11684694 |
|  | 11823472 |
|  | 15546878 |
|  | 11044439 |
|  | 12477932 |
|  | 12522145 |
|  | 10838075 |
|  | 10349636 |
|  | 14581471 |
|  | 10509564 |
|  | 8632902 |
|  | 14871928 |
|  | 11101507 |
|  | 11872750 |
|  | 16162927 |
|  | 8621729 |
|  | 9351825 |
|  | 12556561 |
|  | 11822869 |
|  | 11741894 |
|  | 12522135 |
|  | 12682854 |
|  | 8939914 |
|  | 15888452 |
|  | 11948406 |
|  | 10848576 |
|  | 15299019 |
|  | 17101779 |

---

|  |  |
| --- | --- |
| CellType | hematopoietic cell |
|  | endometrial stromal cell |
|  | squamous cell |
|  | airway epithelial cell |
|  | trophoblast |
|  | germinal center B-cell |
|  | muscle cell |
|  | Purkinje cell |
|  | neuroglia |
|  | epidermis cell |
|  | Leydig cell |
|  | neuron |
|  | ovary follicle cell |
|  | kidney tubule cell |
|  | exocrine cell |
|  | transitional epithelial cell |
|  | smooth muscle myocyte |
|  | hepatocyte |
|  | decidual cell |
|  | macrophage |
|  | pneumocyte |
|  | glandular cells |
|  | lymphoid tissue |
|  | hematopoietic cells |
|  | cells in endometrial stroma |
|  | squamous epithelial cells |
|  | respiratory epithelial cells |
|  | cells in white pulp |
|  | cells in granular layer |
|  | trophoblastic cells |
|  | cells in molecular layer |
|  | germinal center cells |
|  | myocytes |
|  | Purkinje Cells |
|  | cells in seminiferus ducts |
|  | non-germinal center cells |
|  | glial cells |
|  | epidermal cells |
|  | Leydig Cells |
|  | neuronal cells |
|  | cells in glomeruli |
|  | follicle cells |
|  | cells in tubules |
|  | ovarian stroma cells |
|  | exocrine glandular cells |
|  | urothelial cells |
|  | smooth muscle cells |
|  | islets of Langerhans |
|  | bile duct cells |
|  | cells in red pulp |
|  | Hepatocytes |
|  | decidual cells |
|  | Macrophages |
|  | Pneumocytes |

---

|  |  |
| --- | --- |
| Tissue | myocardium |
|  | mouth mucosa |
|  | skeletal muscles |
|  | smooth muscle |
|  | lymphoid tissue |
|  | colon |
|  | Appendix |
|  | duodenum |
|  | bone marrow |
|  | epididymis |
|  | breast |
|  | uterus, post-menopause |
|  | esophagus |
|  | bronchus |
|  | spleen |
|  | fallopian tube |
|  | Cerebellum |
|  | uterus, pre-menopause |
|  | stomach, lower |
|  | Placenta |
|  | gall bladder |
|  | stomach, upper |
|  | Prostate |
|  | lymph node |
|  | heart muscle |
|  | vagina |
|  | testis |
|  | rectum |
|  | hippocampus |
|  | cerebral cortex |
|  | vulva/anal skin |
|  | Salivary gland |
|  | nasopharynx |
|  | thyroid gland |
|  | seminal vesicle |
|  | oral mucosa |
|  | Kidney |
|  | tonsil |
|  | skeletal muscle |
|  | Ovary |
|  | Skin |
|  | lateral ventricle |
|  | small intestine |
|  | Pancreas |
|  | urinary bladder |
|  | Liver |
|  | parathyroid gland |
|  | Lung |
|  | cervix, uterine |
|  | Adrenal gland |

---

|  |  |
| --- | --- |
| Organism | Homo sapiens {Organism urn:agi-taxid:9606} |
|  | Mus musculus {Organism urn:agi-taxid:10090} |
|  | Rattus norvegicus {Organism urn:agi-taxid:10116} |
|  | Homo sapiens |
|  | Mus musculus |
|  | Rattus norvegicus |
|  | Danio rerio |
|  | Bos taurus |
|  | Human |

---

|  |  |
| --- | --- |
| Mouse chromosome position | 9 34.55 cM |
|  | 9 36.0 cM |

---

|  |  |
| --- | --- |
| OMIM ID | 176872 |
|  | 615279 |
|  | 163950 |
|  | 115150 |
|  | OMIM:176872 |
|  | 61385 |

---

|  |  |
| --- | --- |
| Rat chromosome position | 8q24 |

---

|  |  |
| --- | --- |
| Hugo ID | 6840 |
|  | HGNC:6840 |

---

|  |  |
| --- | --- |
| Human chromosome position | 15q22.31 |
|  | 15q22.1-q22.33 |

---

|  |  |
| --- | --- |
| Swiss-Prot Accession | A4QPA9 |
|  | Q02750 |
|  | B4DFY5 |
|  | H3BRW9 |
|  | Q02750.2 |
|  | P31938 |
|  | Q3TMJ8 |
|  | P31938.2 |
|  | Q01986 |
|  | Q01986.2 |
|  | Q5EBD5 |
|  | Q3TMW4 |
|  | Q3U3F4 |
|  | Q3UK05 |
|  | Q9JJE1 |
|  | MP2K1\_HUMAN |
|  | MP2K1\_MOUSE |
|  | MP2K1\_RAT |
|  | Q3TMJ8\_MOUSE |
|  | A4QPA9\_HUMAN |

---

|  |  |
| --- | --- |
| PIR ID | A45100 |
|  | I59571 |
|  | JN0840 |

---

|  |  |
| --- | --- |
| GenBank ID | NC\_000015 |
|  | NM\_002755 |
|  | NP\_002746 |
|  | XM\_017022411 |
|  | XP\_016877900 |
|  | XM\_011521783 |
|  | XP\_011520085 |
|  | XM\_017022412 |
|  | XP\_016877901 |
|  | XM\_017022413 |
|  | XP\_016877902 |
|  | NG\_008305 |
|  | AC011276 |
|  | AC055855 |
|  | AC116913 |
|  | AF190498 |
|  | CH471082 |
|  | EAW77765 |
|  | EAW77766 |
|  | EAW77767 |
|  | EAW77768 |
|  | EAW77769 |
|  | AK022276 |
|  | AK291500 |
|  | BAF84189 |
|  | AK294322 |
|  | BAG57596 |
|  | AK312356 |
|  | BC137459 |
|  | AAI37460 |
|  | BC139729 |
|  | AAI39730 |
|  | BE856741 |
|  | BG709050 |
|  | CA419665 |
|  | DA653983 |
|  | L05624 |
|  | AAA36318 |
|  | L11284 |
|  | Q02750 |
|  | NC\_000075 |
|  | XM\_006511196 |
|  | XP\_006511259 |
|  | NM\_008927 |
|  | NP\_032953 |
|  | AC160118 |
|  | AY902345 |
|  | AAY21055 |
|  | CH466522 |
|  | EDL26058 |
|  | AB041567 |
|  | BAA95051 |
|  | AK146233 |
|  | BAE26999 |
|  | AK146931 |
|  | BAE27542 |
|  | AK154793 |
|  | BAE32832 |
|  | AK165664 |
|  | BAE38326 |
|  | AK165894 |
|  | BAE38443 |
|  | AK167336 |
|  | BAE39437 |
|  | AK181307 |
|  | AK190957 |
|  | AK192193 |
|  | AK192210 |
|  | AK193287 |
|  | AK193946 |
|  | AK194551 |
|  | AK196845 |
|  | AK197193 |
|  | AK199082 |
|  | AK199200 |
|  | AK199288 |
|  | AK200800 |
|  | AK203410 |
|  | AK203853 |
|  | AK214848 |
|  | BC051137 |
|  | BC054754 |
|  | AAH54754 |
|  | BY698931 |
|  | L02526 |
|  | AAA39523 |
|  | P31938 |
|  | NC\_005107 |
|  | NM\_031643 |
|  | NP\_113831 |
|  | XM\_017595427 |
|  | XP\_017450916 |
|  | AC\_000076 |
|  | AABR07070404 |
|  | AABR07070405 |
|  | AABR07070406 |
|  | AABR07070407 |
|  | AABR07070408 |
|  | AABR07070409 |
|  | AABR07070410 |
|  | AABR07070411 |
|  | AABR07070412 |
|  | AABR07070413 |
|  | AABR07070414 |
|  | AABR07070415 |
|  | AABR07070416 |
|  | AABR07070417 |
|  | AABR07070418 |
|  | AABR07070419 |
|  | AABR07070420 |
|  | AABR07070421 |
|  | AABR07070422 |
|  | AABR07070423 |
|  | AABR07070424 |
|  | AABR07070425 |
|  | AABR07070426 |
|  | AABR07070427 |
|  | AABR07070428 |
|  | AABR07070429 |
|  | AABR07070430 |
|  | AAHX01055170 |
|  | AAHX01055171 |
|  | AAHX01055172 |
|  | AAHX01055173 |
|  | CH473975 |
|  | EDL95791 |
|  | AI603089 |
|  | BC089772 |
|  | AAH89772 |
|  | D13341 |
|  | BAA02603 |
|  | D14591 |
|  | BAA03441 |
|  | FQ213946 |
|  | X62313 |
|  | CAA44192 |
|  | Z16415 |
|  | CAA78905 |
|  | Q01986 |
|  | NC\_018926 |
|  | AMYH02030645 |
|  | AMYH02030646 |
|  | AMYH02030647 |
|  | AMYH02030648 |
|  | AMYH02030649 |
|  | AC\_000031 |
|  | AAHY01078835 |
|  | AAHY01078836 |
|  | AAHY01078837 |
|  | AAHY01078838 |
|  | AAHY01078839 |
|  | AAHY01078840 |
|  | XM\_005254541 |
|  | XP\_005254598 |
|  | XM\_006720608 |
|  | XP\_006720671 |
|  | AC\_000147 |
|  | ABBA01038819 |
|  | ABBA01038820 |
|  | ABBA01038821 |
|  | ABBA01038822 |
|  | XM\_006511197 |
|  | XP\_006511260 |
|  | XM\_005254540 |
|  | XP\_005254597 |
|  | AABR06055284 |
|  | AABR06055285 |
|  | NT\_010194 |
|  | NW\_001838218 |
|  | NW\_925884 |
|  | AC\_000058 |
|  | CR604502 |
|  | CR621481 |
|  | NT\_039474 |
|  | NW\_001030907 |
|  | Q3TMJ8 |
|  | Q3TMW4 |
|  | Q3U3F4 |
|  | Q3UK05 |
|  | Q9JJE1 |
|  | NW\_047799 |
|  | NW\_001084873 |
|  | 59808149 |
|  | 59808148 |
|  | 74191215 |
|  | 220811 |
|  | 266566 |
|  | 220812 |
|  | 199124 |
|  | 199123 |
|  | 37540936 |
|  | BC052120 |
|  | 307183 |
|  | BX927065 |
|  | 74191750 |
|  | 74227056 |
|  | 303803 |
|  | 24475715 |
|  | 82226062 |
|  | AAH97064 |
|  | AU041108 |
|  | AW282020 |
|  | 119598175 |
|  | 56628 |
|  | 119598172 |
|  | 119598171 |
|  | 119598174 |
|  | 56629 |
|  | 119598173 |
|  | 400275 |
|  | 123791419 |
|  | 400274 |
|  | NC\_007308 |
|  | 111308433 |
|  | AAH52120 |
|  | XP\_612526 |
|  | 32451982 |
|  | 32451983 |
|  | 51472784 |
|  | 286228 |
|  | Q5EBD5 |
|  | BC097064 |
|  | 51765023 |
|  | 29835265 |
|  | 29835266 |
|  | 62910170 |
|  | NW\_000353 |
|  | 5579478 |
|  | 188568 |
|  | 188569 |
|  | BC119886 |
|  | 13928886 |
|  | AW281751 |
|  | CAK04706 |
|  | 123796627 |
|  | 148694111 |
|  | 123790854 |
|  | CT863714 |
|  | 34865068 |
|  | AAH51137 |
|  | NW\_001492790 |
|  | 407861 |
|  | 407860 |
|  | 81871107 |
|  | 82188372 |
|  | Q4V947 |
|  | 346266 |
|  | Q80XD2 |
|  | 7670398 |
|  | 7670399 |
|  | NM\_213419 |
|  | 66910416 |
|  | NW\_001512924 |
|  | Q7ZTX6 |
|  | AAI19887 |
|  | 74226698 |
|  | 6678793 |
|  | 6678794 |
|  | 149041950 |
|  | NT\_086827 |
|  | Q0VD16 |
|  | Q1LUY7 |
|  | 14589898 |
|  | 123888458 |
|  | 33859656 |
|  | 148726469 |
|  | 30353821 |
|  | NP\_998584 |
|  | NC\_007113 |
|  | 81917507 |
|  | XM\_612526 |
|  | 141796928 |
|  | 123790130 |
|  | AW281434 |
|  | 74147303 |
|  | 122143526 |
|  | 94733356 |
|  | CAN88104 |
|  | 81909568 |
|  | 74139093 |

---

|  |  |
| --- | --- |
| Swiss-Prot ID | MP2K1\_HUMAN |
|  | MP2K1\_MOUSE |
|  | MP2K1\_RAT |
|  | A4QPA9\_HUMAN |
|  | Q3TMJ8\_MOUSE |

---

|  |  |
| --- | --- |
| Cell Localization | Cytoplasm |
|  | Cytoskeleton |
|  | Microtubule organizing center |
|  | Centrosome |
|  | Spindle pole body |
|  | Nucleus |
|  | Membrane |
|  | Cytoplasm, cytoskeleton, microtubule organizing center, centrosome |
|  | Cytoplasm, cytoskeleton, microtubule organizing center, spindle pole body |

---

|  |  |
| --- | --- |
| Ensembl ID | ENSG00000169032 |
|  | ENSP00000302486.4 |
|  | ENST00000307102.9 |
|  | ENSP00000456438.1 |
|  | ENST00000566326.1 |
|  | ENSMUSG00000004936 |
|  | ENSMUSP00000005066.8 |
|  | ENSMUST00000005066.8 |
|  | ENSRNOG00000010176 |
|  | ENSRNOP00000013933.4 |
|  | ENSRNOT00000013933.6 |
|  | ENSP00000302486 |
|  | ENST00000307102 |
|  | ENSMUSP00000005066 |
|  | ENSMUST00000005066 |
|  | ENSRNOP00000013933 |
|  | ENSRNOT00000013933 |

---

|  |  |
| --- | --- |
| MGI ID | MGI:1346866 |
|  | 1346866 |
|  | 102562 |

---

|  |  |
| --- | --- |
| RGD ID | 70495 |
|  | 61385 |

---

|  |  |
| --- | --- |
| Unigene ID | Hs.145442 |
|  | Mm.248907 |
|  | Rn.5850 |
|  | Mm.435672 |
|  | Mm.426740 |
|  | Mm.465469 |
|  | Dr.81130 |
|  | Mm.456154 |
|  | Bt.29185 |
|  | Hs.132311 |
|  | Dr.107100 |

---

|  |  |
| --- | --- |
| Homologene ID | 2063 |

---

|  |  |
| --- | --- |
| Shape | Sickle-vertex |

---

|  |  |
| --- | --- |
| IPI ID | IPI00219604 |
|  | IPI00797850 |
|  | IPI00231247 |
|  | IPI00466610 |

---

|  |  |
| --- | --- |
| Microarray ID | L11284\_at |
|  | l04485mrna\_s\_at |
|  | 1844\_s\_at |
|  | MB170 |
|  | 335 |
|  | d14591\_s\_at |
|  | l02526\_s\_at |
|  | 202670\_at |
|  | 92585\_at |
|  | 1416351\_at |
|  | OR0879 |
|  | A\_14\_P100756 |
|  | rc\_ai178835\_at |
|  | A\_42\_P638191 |
|  | A\_14\_P118260 |
|  | R724 |
|  | A\_23\_P20248 |
|  | A\_51\_P241074 |
|  | 60945\_at |
|  | 1130\_at |
|  | L05624\_s\_at |
|  | OH7696 |
|  | 99707\_at |
|  | A\_32\_P189110 |
|  | A\_32\_P406652 |
|  | L05624 |
|  | 214592\_s\_at |
|  | M347 |
|  | OH7707 |
|  | 1367760\_at |
|  | A\_14\_P128795 |

---

|  |  |
| --- | --- |
| Homo sapiens Chromosome position | 15q22.1-q22.33 |

---

|  |  |
| --- | --- |
| Ensembl Transcript ID | ENST00000307102 |

---

|  |  |
| --- | --- |
| ENSEMBL | ENSG00000169032 |

---

|  |  |
| --- | --- |
| FunctionalClass | MAP kinase kinase |
|  | kinase |
|  | protein kinase |
|  | protein binding |
|  | protein-tyrosine kinase |
|  | nucleotide binding |
|  | protein serine/threonine kinase |
|  | transferase |
|  | ATP binding |

---

|  |  |
| --- | --- |
| Hugo Symbol | MAP2K1 |

---

|  |  |
| --- | --- |
| Bos taurus Chromosome position | 10 |

---

|  |  |
| --- | --- |
| Mus musculus Chromosome position | 9 36.0 cM |

---

|  |  |
| --- | --- |
| ProteinAtlas SubcellularLocation | Main location: Plasma membrane;Cytoplasm; Other location: ; Expression type: Staining; Reliability: Supportive |

---

|  |  |
| --- | --- |
| Rattus norvegicus Chromosome position | 8q24 |

---

|  |  |
| --- | --- |
| Ensembl Gene ID | ENSG00000169032 |

---

|  |  |
| --- | --- |
| ProteinAtlas NormalTissue | Tissue: nasopharynx; Cell type: respiratory epithelial cells; Level: Moderate; Expression type: Staining; Reliability: Uncertain |
|  | Tissue: duodenum; Cell type: glandular cells; Level: Weak; Expression type: Staining; Reliability: Uncertain |
|  | Tissue: uterus |
|  | post-menopause; Cell type: glandular cells; Level: Weak; Expression type: Staining; Reliability: Uncertain |
|  | Tissue: spleen; Cell type: cells in white pulp; Level: Weak; Expression type: Staining; Reliability: Uncertain |
|  | Tissue: spleen; Cell type: cells in red pulp; Level: Moderate; Expression type: Staining; Reliability: Uncertain |
|  | Tissue: oral mucosa; Cell type: squamous epithelial cells; Level: Moderate; Expression type: Staining; Reliability: Uncertain |
|  | Tissue: colon; Cell type: glandular cells; Level: Weak; Expression type: Staining; Reliability: Uncertain |
|  | Tissue: stomach |
|  | lower; Cell type: glandular cells; Level: Moderate; Expression type: Staining; Reliability: Uncertain |
|  | Tissue: pancreas; Cell type: exocrine glandular cells; Level: Strong; Expression type: Staining; Reliability: Uncertain |
|  | Tissue: smooth muscle; Cell type: smooth muscle cells; Level: Negative; Expression type: Staining; Reliability: Uncertain |
|  | Tissue: lung; Cell type: pneumocytes; Level: Low; Expression type: APE; Reliability: High |
|  | Tissue: adrenal gland; Cell type: glandular cells; Level: Medium; Expression type: APE; Reliability: High |
|  | Tissue: appendix; Cell type: glandular cells; Level: Medium; Expression type: APE; Reliability: High |
|  | Tissue: appendix; Cell type: lymphoid tissue; Level: Medium; Expression type: APE; Reliability: High |
|  | Tissue: bone marrow; Cell type: hematopoietic cells; Level: High; Expression type: APE; Reliability: High |
|  | Tissue: breast; Cell type: glandular cells; Level: Medium; Expression type: APE; Reliability: High |
|  | Tissue: bronchus; Cell type: respiratory epithelial cells; Level: Low; Expression type: APE; Reliability: High |
|  | Tissue: cerebellum; Cell type: cells in granular layer; Level: Medium; Expression type: APE; Reliability: High |
|  | Tissue: cerebellum; Cell type: cells in molecular layer; Level: Medium; Expression type: APE; Reliability: High |
|  | Tissue: cerebellum; Cell type: Purkinje cells; Level: High; Expression type: APE; Reliability: High |
|  | Tissue: cerebral cortex; Cell type: glial cells; Level: Low; Expression type: APE; Reliability: High |
|  | Tissue: cerebral cortex; Cell type: neuronal cells; Level: Medium; Expression type: APE; Reliability: High |
|  | Tissue: cervix, uterine; Cell type: glandular cells; Level: Medium; Expression type: APE; Reliability: High |
|  | Tissue: cervix, uterine; Cell type: squamous epithelial cells; Level: Medium; Expression type: APE; Reliability: High |
|  | Tissue: colon; Cell type: glandular cells; Level: Medium; Expression type: APE; Reliability: High |
|  | Tissue: duodenum; Cell type: glandular cells; Level: Medium; Expression type: APE; Reliability: High |
|  | Tissue: epididymis; Cell type: glandular cells; Level: Medium; Expression type: APE; Reliability: High |
|  | Tissue: esophagus; Cell type: squamous epithelial cells; Level: Low; Expression type: APE; Reliability: High |
|  | Tissue: fallopian tube; Cell type: glandular cells; Level: Low; Expression type: APE; Reliability: High |
|  | Tissue: gall bladder; Cell type: glandular cells; Level: Medium; Expression type: APE; Reliability: High |
|  | Tissue: heart muscle; Cell type: myocytes; Level: None; Expression type: APE; Reliability: High |
|  | Tissue: hippocampus; Cell type: glial cells; Level: Low; Expression type: APE; Reliability: High |
|  | Tissue: hippocampus; Cell type: neuronal cells; Level: Medium; Expression type: APE; Reliability: High |
|  | Tissue: kidney; Cell type: cells in glomeruli; Level: Low; Expression type: APE; Reliability: High |
|  | Tissue: kidney; Cell type: cells in tubules; Level: Medium; Expression type: APE; Reliability: High |
|  | Tissue: lateral ventricle; Cell type: glial cells; Level: Low; Expression type: APE; Reliability: High |
|  | Tissue: lateral ventricle; Cell type: neuronal cells; Level: Medium; Expression type: APE; Reliability: High |
|  | Tissue: liver; Cell type: bile duct cells; Level: Medium; Expression type: APE; Reliability: High |
|  | Tissue: liver; Cell type: hepatocytes; Level: None; Expression type: APE; Reliability: High |
|  | Tissue: lung; Cell type: macrophages; Level: Medium; Expression type: APE; Reliability: High |
|  | Tissue: lymph node; Cell type: germinal center cells; Level: Medium; Expression type: APE; Reliability: High |
|  | Tissue: lymph node; Cell type: non-germinal center cells; Level: Low; Expression type: APE; Reliability: High |
|  | Tissue: nasopharynx; Cell type: respiratory epithelial cells; Level: Medium; Expression type: APE; Reliability: High |
|  | Tissue: oral mucosa; Cell type: squamous epithelial cells; Level: Low; Expression type: APE; Reliability: High |
|  | Tissue: ovary; Cell type: follicle cells; Level: Low; Expression type: APE; Reliability: High |
|  | Tissue: ovary; Cell type: ovarian stroma cells; Level: Low; Expression type: APE; Reliability: High |
|  | Tissue: pancreas; Cell type: exocrine glandular cells; Level: Medium; Expression type: APE; Reliability: High |
|  | Tissue: pancreas; Cell type: islets of Langerhans; Level: Low; Expression type: APE; Reliability: High |
|  | Tissue: parathyroid gland; Cell type: glandular cells; Level: Medium; Expression type: APE; Reliability: High |
|  | Tissue: placenta; Cell type: decidual cells; Level: Medium; Expression type: APE; Reliability: High |
|  | Tissue: placenta; Cell type: trophoblastic cells; Level: Medium; Expression type: APE; Reliability: High |
|  | Tissue: prostate; Cell type: glandular cells; Level: Medium; Expression type: APE; Reliability: High |
|  | Tissue: rectum; Cell type: glandular cells; Level: Medium; Expression type: APE; Reliability: High |
|  | Tissue: salivary gland; Cell type: glandular cells; Level: Low; Expression type: APE; Reliability: High |
|  | Tissue: seminal vesicle; Cell type: glandular cells; Level: Low; Expression type: APE; Reliability: High |
|  | Tissue: skeletal muscle; Cell type: myocytes; Level: None; Expression type: APE; Reliability: High |
|  | Tissue: skin; Cell type: epidermal cells; Level: Low; Expression type: APE; Reliability: High |
|  | Tissue: small intestine; Cell type: glandular cells; Level: Medium; Expression type: APE; Reliability: High |
|  | Tissue: smooth muscle; Cell type: smooth muscle cells; Level: None; Expression type: APE; Reliability: High |
|  | Tissue: spleen; Cell type: cells in red pulp; Level: Medium; Expression type: APE; Reliability: High |
|  | Tissue: spleen; Cell type: cells in white pulp; Level: Low; Expression type: APE; Reliability: High |
|  | Tissue: stomach, lower; Cell type: glandular cells; Level: Medium; Expression type: APE; Reliability: High |
|  | Tissue: stomach, upper; Cell type: glandular cells; Level: Medium; Expression type: APE; Reliability: High |
|  | Tissue: testis; Cell type: cells in seminiferus ducts; Level: Medium; Expression type: APE; Reliability: High |
|  | Tissue: testis; Cell type: Leydig cells; Level: Medium; Expression type: APE; Reliability: High |
|  | Tissue: thyroid gland; Cell type: glandular cells; Level: Low; Expression type: APE; Reliability: High |
|  | Tissue: tonsil; Cell type: germinal center cells; Level: High; Expression type: APE; Reliability: High |
|  | Tissue: tonsil; Cell type: non-germinal center cells; Level: Medium; Expression type: APE; Reliability: High |
|  | Tissue: tonsil; Cell type: squamous epithelial cells; Level: High; Expression type: APE; Reliability: High |
|  | Tissue: urinary bladder; Cell type: urothelial cells; Level: Medium; Expression type: APE; Reliability: High |
|  | Tissue: uterus, post-menopause; Cell type: cells in endometrial stroma; Level: None; Expression type: APE; Reliability: High |
|  | Tissue: uterus, post-menopause; Cell type: glandular cells; Level: Low; Expression type: APE; Reliability: High |
|  | Tissue: uterus, pre-menopause; Cell type: cells in endometrial stroma; Level: Low; Expression type: APE; Reliability: High |
|  | Tissue: uterus, pre-menopause; Cell type: glandular cells; Level: Medium; Expression type: APE; Reliability: High |
|  | Tissue: vagina; Cell type: squamous epithelial cells; Level: Low; Expression type: APE; Reliability: High |
|  | Tissue: vulva/anal skin; Cell type: epidermal cells; Level: Low; Expression type: APE; Reliability: High |

---

|  |  |
| --- | --- |
| LocatorString | pmftq:achondroplasia |
|  | gscquery:achondroplasia |

---
